# Developing ANDI: A Novel Approach to Health Product R&D in Africa

**DOI:** 10.1371/journal.pmed.1000293

**Published:** 2010-06-29

**Authors:** Solomon Nwaka, Tshinko B. Ilunga, Jorge Santos Da Silva, Emiliano Rial Verde, Doan Hackley, Raymond De Vré, Tom Mboya-Okeyo, Robert G. Ridley

**Affiliations:** 1 UNICEF/UNDP/World Bank/WHO Special Programme for Research and Training in Tropical Diseases (TDR), World Health Organization, Geneva, Switzerland; 2 African Development Bank, Tunis-Belvedère, Tunisia; 3 McKinsey & Company, Geneva, Switzerland; 4 Permanent Mission of the Republic of Kenya to the United Nations and Other International Organizations, Geneva, Switzerland

## Abstract

Solomon Nwaka and colleagues discuss ANDI, the African Network for Drugs and Diagnostics Innovation, which is intended to help stimulate health research and development on the African continent.

**Summary Points**

- A novel approach to supporting health product research and development (R&D) and access in Africa is urgently needed. Successful implementation of such an endeavor requires sustainable capacity, infrastructure, funding, leadership, and an understanding of the status of health R&D in the African continent.
- As part of the development of the African Network for Drugs and Diagnostics Innovation (ANDI), we analyzed biomedical research output and collaborative research undertaken across the continent by evaluating peer-reviewed articles published between 2004 and 2008, as well as other innovation indicators, such as R&D investments and manufacturing capacity.
- Significant health R&D capacity exists in different parts of Africa, but this capacity is fragmented, uncoordinated, and not properly utilized to address African health problems. Most biomedical collaborations of African institutions are with institutions in Europe and the United States rather than with other African institutions. This lack of intracontinental collaboration, combined with low levels of investment, contributes to gaps in the continental research agenda, a lack of local ownership of research undertaken on the continent, and suboptimal utilization of available research capability.
- We discuss the establishment of ANDI as a new approach to address these challenges, through the creation, coordination, and funding of African health R&D networks focused on the discovery, development, and delivery of tools to address Africa's unique health needs.

## A Need for African-Led Health R&D Innovation

The health status of the African population remains behind that of populations in Europe and North America, as well as many other developing regions with similar affluence (Figure S1). For example, Africa is especially affected by a series of infectious diseases that are responsible for more than half of its disability-adjusted life years (DALYs) and over 6 million deaths per year (Figure S2). For the 18 diseases listed in Figure S2 Africa has over 30%, and in some cases over 90%, of the worldwide disease burden, even though it represents only 15% of the global population. Currently, there are limited or no affordable therapies or vaccines for many of these conditions, and diagnostic methods, where they exist, are often inadequate to deploy in the field for large populations [1]–[5]. Accurate quantification of the economic impact of disease burden is difficult. However, the negative impact of these diseases to the African gross domestic product (GDP) may run into tens of billions of dollars (US) each year [5]–[7].

**Figure 1:**
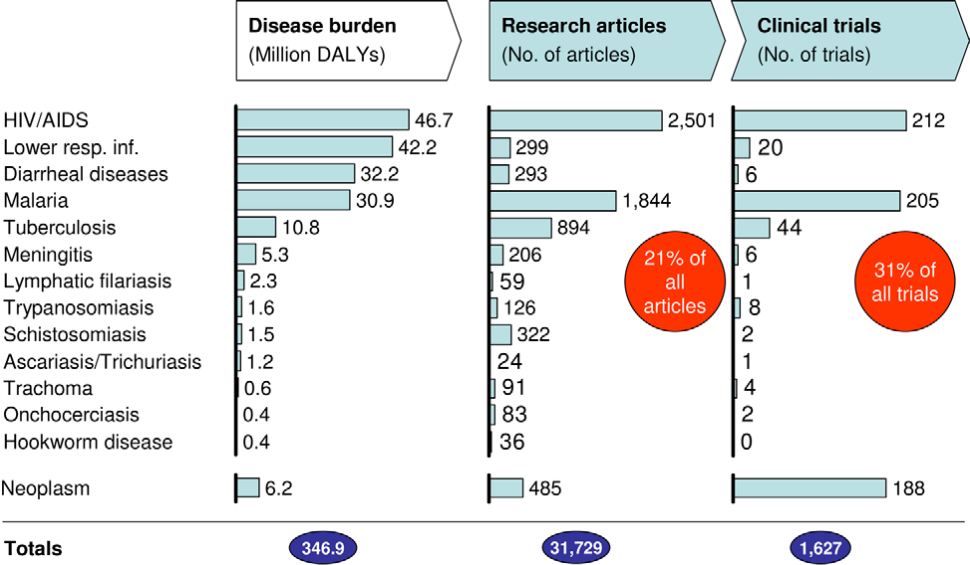
Diseases disproportionately affecting Africa are under-prioritized. Only 21% of all articles with at least one African author focus on the conditions causing highest disease burden in Africa. This reduced research focus mirrors the low number of products being tested in clinical trials (only ∼1/3 of trials). African DALYs were obtained from the Global Burden of Disease project of WHO. Peer-reviewed articles with at least one African author published in the biomedical fields during the 5-year period between 2004 and 2008 were identified by querying the Thomson Web of Science database for African countries in the affiliation field (Figure 2 and Text S1). Absolute numbers of clinical trials were identified using the ClinicalTrials.gov database (as of September 2009). All trials for drugs and biological products with at least one center in Africa were considered and all 53 focus countries were scanned. Only trials “currently open” or “recently completed” (i.e., concluded within 48 months of scan date) were counted. A total of 1,627 trials were identified, out of which 511 trials focus on drugs or biologicals for diseases identified as causing the highest disease burden in Africa (see left column of charts).

Despite some welcome increases in global R&D investment in recent years (e.g., through public private partnerships), it is generally agreed that research and product pipelines for the diseases that disproportionately affect developing countries are grossly inadequate. This has recently been reinforced by the 192 countries composing the World Health Assembly through resolutions supporting GSPOA (the Global Strategy and Plan of Action on Public Health, Innovation and Intellectual Property) [8],[9]. There is urgent need for enhanced research in developing countries. Our analysis suggest that, in addition to the scant research for several diseases, only 21% of total biomedical research publications and 31% of clinical trials in Africa are related to diseases that account for approximately 50% of the African disease burden (Figure 1).

One of the underlying emphases of the GSPOA was that a sustainable global solution requires greater efforts to build and utilize innovation capabilities in developing countries, enhance their access to information and technology, and forge collaborative networks. Implementing these goals and a sustainable pan-African health product R&D endeavor requires capacity, infrastructure, leadership, financing, and an understanding of the status of health R&D in the African continent. In a related development, the African Union have committed to raising spending on scientific research and innovation to 1% of GDP in recognition that such funding is a necessary prerequisite for sustainable development [10]. We therefore assessed the African biomedical research landscape including the level of intra-African expertise and collaboration to support the development of the African Network for Drug and Diagnostics Innovation (ANDI) in contributing to the implementation of these goals.

## Health R&D in Africa: Variable Capacity and Lack of Intra-African Collaboration

**Figure 2:**
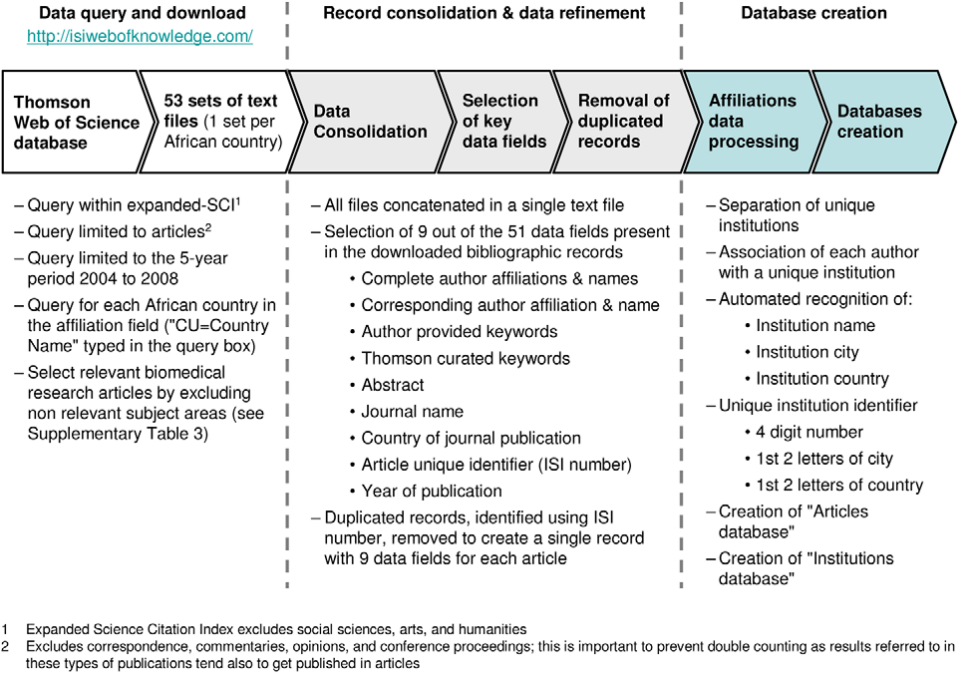
Flow chart: Creation of Bibliographic databases.

We mapped the African health research landscape by building a database of all peer-reviewed research articles in biomedical fields that had at least one African-based author, during the 5-year period 2004 to 2008. The methodology for data collection and analysis is presented in Figure 2 (additional information is also provided in Text S1 and Table S3). The affiliation of authors in a total of 31,279 articles identified were processed to determine the lead and collaborating institutions in each article. For every institution, the number of individual collaborations was quantified and mapped.

**Figure 3:**
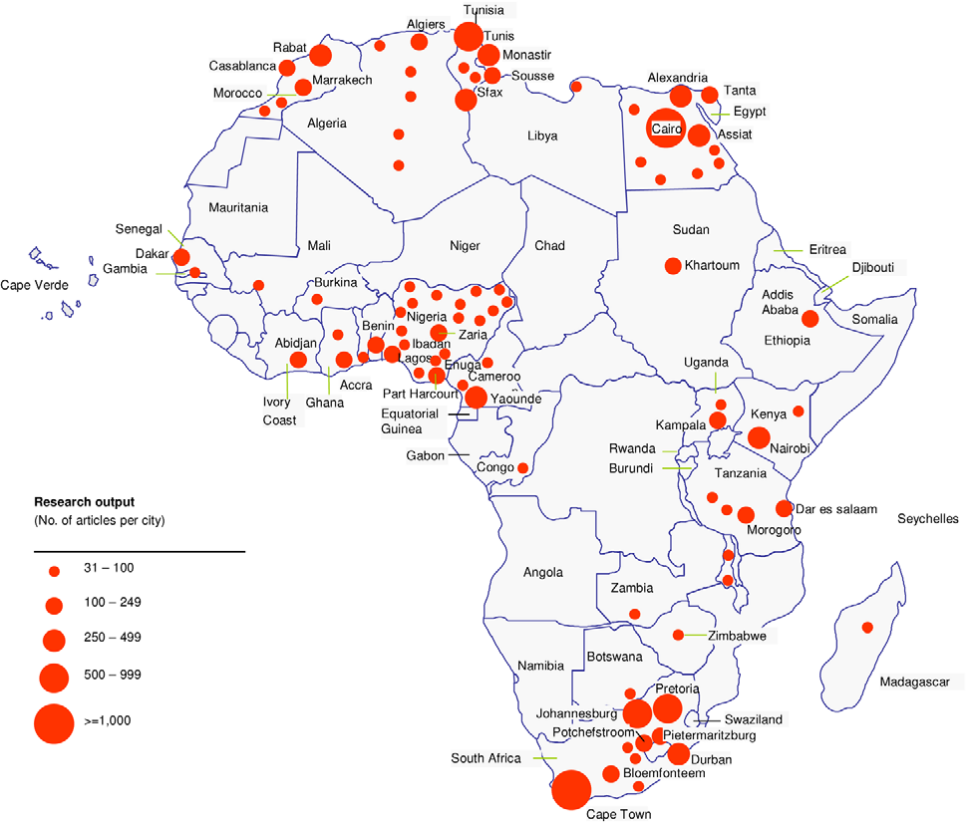
Distribution of R&D capacity in Africa. Mapping of the top African cities by research output shows hotspots of R&D activity, but also highlights inequities in R&D productivity across the continent. To create this mapping we used articles with Africa-based authors in the corresponding address. A total of 20,119 articles were identified (Figure 2 and Text S1), but for simplicity only African cities with over 30 articles from 2004 to 2008 are marked. This represents 16,647 articles (circle diameter indicates number of published articles; a total of 91 cities in 28 countries are identified but only the top 40 are labeled).

We identified about 2,700 institutions in Africa as lead institutions based on the fact that they were the corresponding institutions for articles cited in the peer-reviewed article database. These are present in 47 of the 53 African countries (excluding Cape Verde, Equatorial Guinea, Liberia, São Tomé and Principe, Somalia, and Burundi). This significant number indicates that quality R&D capacity exists in the continent. Figure 3 illustrates centres of expertise with more than 30 biomedical publications. Concurrently, mapping of clinical trial activity in Africa also highlights the existence of significant capacity, as well as pharmaceutical manufacturing capacity with over 120 companies identified and some patent activity (data not shown). However, the data also highlight the challenging reality that distribution of R&D capacity is uneven in Africa. These findings are consistent with, but go beyond, an earlier report on product R&D landscape in Africa [11],[12].

Among the top 20 most productive institutions, i.e., those with the highest number of articles published, we found that only three African countries are represented (South Africa, Egypt, and Nigeria). This analysis show that portions of Western and Central Africa are significantly lagging behind (Figure 3). This trend is further confirmed by patent productivity, which is concentrated in a few countries in Africa (data not shown). These patterns underscore the value of increasing collaboration across African countries to both increase and leverage the available expertise to enhance R&D capacity.

**Figure 4:**
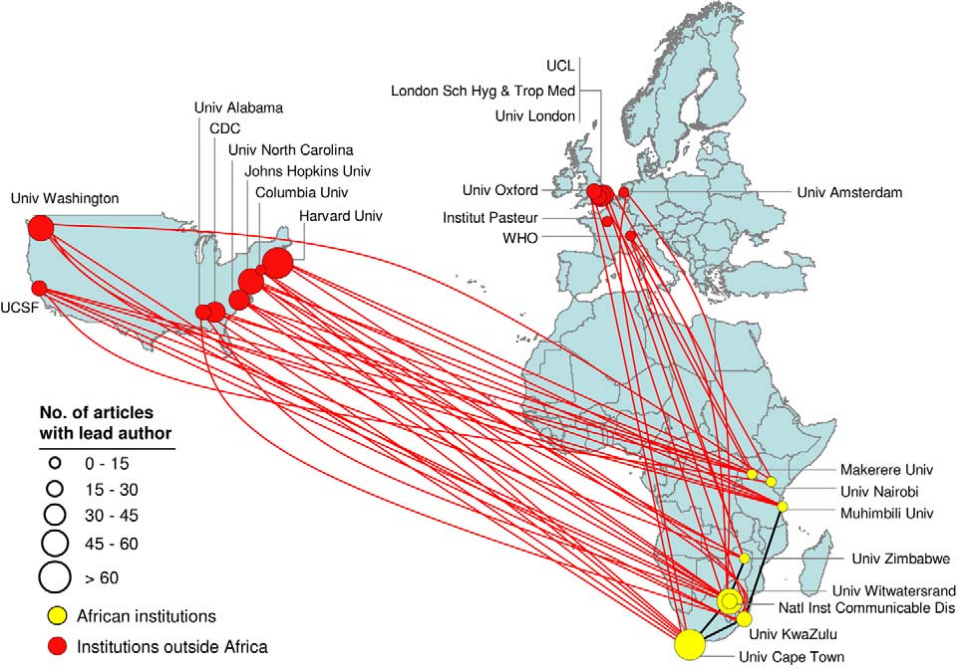
Collaboration bias towards the US and Europe for the HIV/AIDS R&D network. Network mapping of most collaborative institutions leading articles identified as directly related to HIV/AIDS and published from 2004 to 2008. There are eight top centers in Africa, mostly in the Southern region. Few articles are published in collaboration between these African centers but there is active collaboration with institutions in the Northern Hemisphere, mainly in the US. Yellow circles indicate institutions in Africa and red circles mark institutions outside of Africa. Circle diameter indicates the count of HIV/AIDS articles identified in the analyzed period. Only the top most collaborative institutions in the network and the links to and from Africa among them are shown. Institutions outside Africa that do not show connections (e.g., Institut Pasteur) are linked to other institutions outside Africa or to institutions not identified as the most collaborative.

We next assessed the nature of collaborative activity by African institutions. While 77% of articles in our database are authored in collaboration, only 5.4% involve institutions in more than one African country, and fewer than 1% involve more than two African countries. The vast majority of collaborations are with external partners in Europe and the United States. Even the most collaborative African institutions have little collaboration with African countries other than their own (Table S1). This further confirms the low degree of intra-African R&D collaboration. The focus of collaboration with the United States and European countries is illustrated by the R&D network mapping for two critical diseases disproportionately affecting Africa: HIV/AIDS and malaria (Figures 4 and S3). These figures identify the top 20 city nodes of collaboration using measures of “degree” and “betweenness” for each disease and mapping their main collaborative partners (see Text S1). These measures are commonly used as measures of network centrality [13]–[17]. Other diseases disproportionately affecting Africa show a similar pattern (Table S2).

A closer look at the HIV/AIDS and malaria network maps reveals some subtle differences in relation to these two diseases: (a) there are significantly more research articles for HIV/AIDS than for malaria, but the percentage of African collaboration is approximately twice as high for malaria (13%) as for HIV/AIDS (7%) (Table S2); (b) malaria collaborations are more widely spread across Africa than they are for HIV/AIDS; (c) HIV/AIDS research more strongly partners with the US, while malaria research more strongly partners with Europe. A clear explanation for these differences will require more work. However, we believe that the higher collaboration seen for malaria is a reflection of a longer history of local malaria research in Africa.

## Implications of Data for the Development of African-Led Health R&D Innovation

While the extra-African collaboration should be encouraged, the lack of intra-African collaboration suggests that African institutions do not have adequate leadership and ownership of the research being done in the continent. The sustainability of research undertaken in Africa may also be an issue, especially when it is undertaken with short-term funds coming from, and directed from, external sources. The poor intra-African collaboration was confirmed by interviews in Africa. Over 170 stakeholders were interviewed across African countries/regions including ministries of health, science and technology, and higher education; national academies of science; pharmaceutical companies and research centers; and networks and universities in South Africa, Nigeria, Egypt, Kenya, and Burkina Faso [6]. These interviews identified key factors believed to hamper collaboration, ownership, and leadership of research in Africa. These include the lack of knowledge about R&D done in other African countries, the deficient networking infrastructure, and the absence of financial incentives to spur cooperative research within the continent.

Increasing R&D activities for under-researched diseases and promoting collaborative networks within Africa will require robust African-based funding mechanisms to complement current funding that is coming mostly from outside Africa. Based on data from the UNESCO Science Report 2005, Africa spent 0.3% of GDP on R&D in 2002, in contrast to a global value of 1.7%. Increased local funding and intra-African coordination to complement external support and coordination are essential to spur much-needed health R&D and empower Africa in driving its own R&D agenda. Overdependence on external sources alone will continue to leave a substantial portion of Africa health needs unaddressed, and will not resolve the leadership and ownership gap. The implementation of ANDI is hoped to help in addressing these challenges.

## The African Network for Drugs and Diagnostics Innovation

The global momentum to increase participation and leadership of low-income countries in their own R&D programs received a major push through World Health Assembly resolutions on the Global Strategy and Plan of Action on Public Health, Innovation and Intellectual Property [8],[9]. This guiding framework calls for improvement in health R&D innovation through greater engagement of developing countries, investment in local capacity and capability building including support for regional R&D collaborative networks, and devising mechanisms to ensure financial sustainability of local R&D efforts. This is consistent with other high-level pan-African declarations, for example the Algiers declaration of 2008 [18]. This is a propitious moment to address the need for medicines in Africa, with a pragmatic and sustainable model that: (a) promotes the assembly of African R&D networks that can better use the technology and human capital already present on the Continent, (b) sustainably funds R&D projects aligned with African health priorities and led by African R&D centers, (c) ensures African ownership of the R&D agenda, and (d) supports broader south–south and south–north collaboration and technology transfer.

A strategic business plan to guide the implementation and financing of drugs and diagnostics research—ANDI—has been developed by a series of partners such as WHO through TDR, AFRO (WHO African Regional Office), and EMRO (WHO Eastern Mediterranean Regional Office), the African Development Bank, the European Union, and several national African institutions [6]. The plan calls for the establishment of an African innovation fund to support ANDI activities. Discussions are ongoing to formally establish an African-led governance structure for ANDI under the auspices of an African institution in 2010, and to operationally launch the initiative with a set of well-defined projects in 2011.

The business plan's development involved multiple consultative discussions, analyses, and over 170 stakeholder interviews. The plan calls for a US$600 million endowment fund in Africa that can complement other, more classical, donations to generate a sustainable income of up to US$30 million annually to support African health product innovation. ANDI aims to partner, fund, and coordinate research by creating a portfolio of collaborative project networks and partnerships as well as building capacity and support for infrastructural development. It will also advocate for more investment for research to be done throughout Africa and support local intellectual property management capability for enhanced access to medicines. Recent discussions with various stakeholders have also emphasized the need for ANDI to remain open to include research on a broad range of products, including drugs, diagnostics, vaccines, and medical devices. They have also stressed the need for downstream research to strengthen and support health systems.

ANDI will complement external R&D efforts, such as those promoted by product development partnerships that focus on one or a few diseases. These include, Medicines for Malaria Venture (www.mmv.org), Drugs for Neglected Diseases Initiative (www.dndi.org), Foundation for Innovative New Diagnostics (www.finddiagnostics.org) and Malaria Vaccine Initiative (www.malariavaccine.org/) [19]. It will also complement and partner other continental initiatives such as EDCTP (European and Developing Countries Clinical Trials Partnership, http://www.edctp.org/), AMANET (African Malaria Network Trust, http://www.amanet-trust.org/), and AAVP (African AIDS Vaccine Programme, http://www.who.int/vaccine_research/diseases/hiv/aavp/en/) [20], which focus primarily on clinical research. It will also complement capacity-building efforts such as those of the Wellcome Trust focusing on academic biomedical research capacity in specific countries such as Kenya and Malawi. ANDI covers the entire innovation value chain from discovery to manufacture and links both health and innovation sectors to economic development.

Funds have been secured, including from the European Union, to support the initial establishment of ANDI. However, further resources are being sought to operationalise it in Africa. Next steps for the establishment of ANDI include: (a) the formal establishment of a governance structure; (b) the selection of host sites for the regional and sub-regional offices; (c) the selection and establishment of an initial set of projects and technological support platforms; and (d) recruitment of staff. It is anticipated that these milestones will be achieved by 2011. Progress towards these activities will be reported at the 3rd ANDI stakeholders' meetings scheduled for Nairobi in October 2010.

The establishment of ANDI as a functional and successful organization located in Africa—managed and governed by African institutions, implementing product R&D, and ensuring sustainable access to new drugs and diagnostics innovations—will help fill the gaps identified in this work, including deficient investment in product R&D within Africa; a lack of collaboration among African scientists and between the African public and private sector; and poor awareness of the link between research and economic development.

## Supporting Information

Figure S1 Life expectancy in Africa. Life expectancy in Africa is in general lower than in other areas of the developing world (e.g., Asia). This is independent of economic development, as measured by GDP per capita. GDP per capita is shown in a logarithmic scale, and correspond to purchasing power parity (PPP) in 2007. All measurements obtained from the United Nations [5].
(0.16 MB TIF)

Figure S2 Disease burden caused by diseases disproportionally affecting Africa. Diseases that show high relative impact in Africa, i.e., cause over 30% of total global disease burden as measured by DALYs. DALY is a time-based measure combining years of life lost to premature mortality and years of life lost to time lived in states of less than full health. These are responsible for 54% of all African DALYs and approximately half of all deaths.
(0.41 MB TIF)

Figure S3 Collaboration bias towards the US and Europe for the malaria R&D network. Network mapping of most collaborative institutions leading articles identified as directly related to malaria and published from 2004 to 2008. There are nine top centers in Africa spread across the continent, except in the northern region. Few articles are published in collaboration between African centers, but these are active in collaborating with institutions in the Northern Hemisphere, mainly in Europe. Yellow circles indicate institutions in Africa; red circles mark institutions outside of Africa. Circle diameter indicates the count of malaria articles identified in the analyzed period. Only the top most-collaborative institutions in the network and the links to and from Africa among them are shown.
(0.55 MB TIF)

Table S1 Most-collaborative African institutions.
(0.41 MB TIF)

Table S2 African R&D output in eight key disease areas in Africa.
(0.18 MB TIF)

Table S3 Subject areas excluded to restrict the dataset to biomedical publications.
(0.72 MB TIF)

Text S1 Methodology.
(0.03 MB DOC)

## Abbreviations

ANDI: African Network for Drugs and Diagnostics Innovation
AMANET: African Malaria Network Trust
AAVP: African AIDS Vaccine Programme
DALY: disability-adjusted life year
EDCTP: European and Developing Countries Clinical Trials Partnership
GDP: gross domestic product
GSPOA: Global Strategy and Plan of Action on Public Health, Innovation and Intellectual Property
R&D: research and development
TDR: UNICEF/UNDP/World Bank/WHO Special Programme for Research and Training in Tropical Diseases
UNESCO: United Nations Educational, Scientific and Cultural Organization
WHO: World Health Organization
AFRO: WHO African Regional Office
EMRO: WHO Eastern Mediterranean Regional Office
