# Supplementary figures and images for: Developing ANDI: A Novel Approach to Health Product R&D in Africa
Source: PLoS Med. 2010 Jun 29;7(6):e1000293. doi: 10.1371/journal.pmed.1000293 (PMC2893959; doi:10.1371/journal.pmed.1000293)

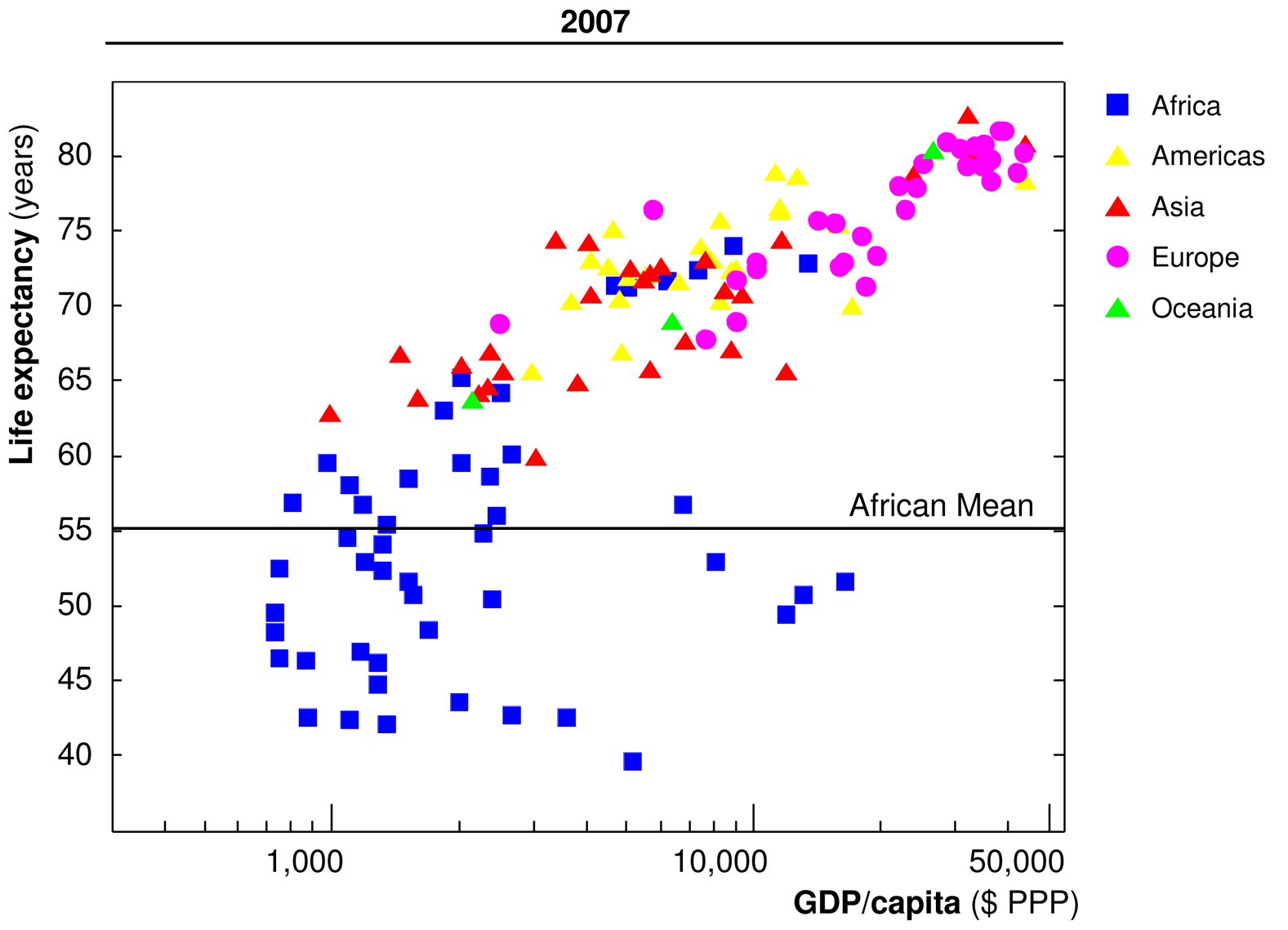

Supplement: Figure S1 — Life expectancy in Africa. Life expectancy in Africa is in general lower than in other areas of the developing world (e.g., Asia). This is independent of economic development, as measured by GDP per capita. GDP per capita is shown in a logarithmic scale, and correspond to purchasing power parity (PPP) in 2007. All measurements obtained from the United Nations [5]. (0.16 MB TIF) [file pmed.1000293.s001.tif]

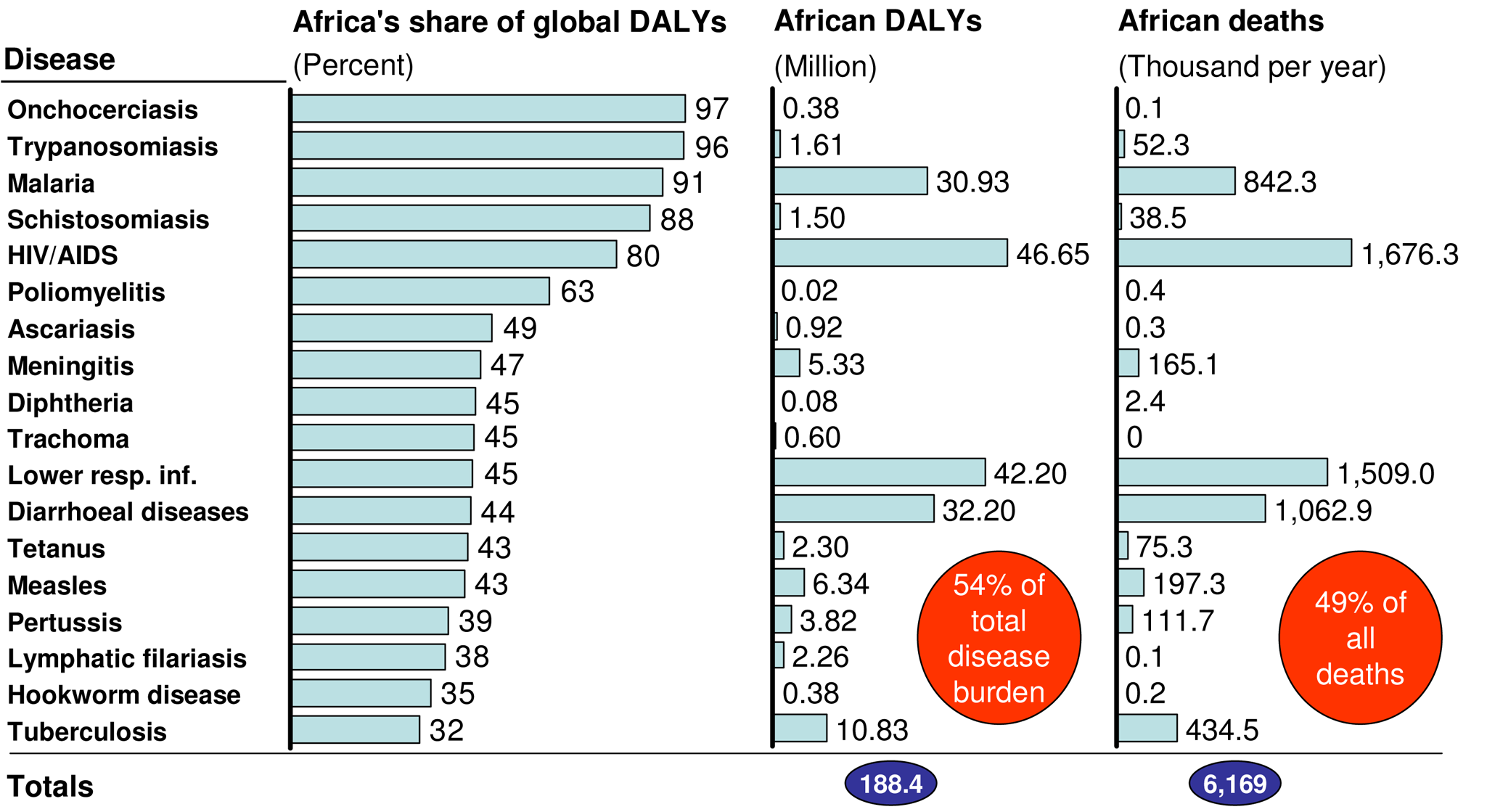

Supplement: Figure S2 — Disease burden caused by diseases disproportionally affecting Africa. Diseases that show high relative impact in Africa, i.e., cause over 30% of total global disease burden as measured by DALYs. DALY is a time-based measure combining years of life lost to premature mortality and years of life lost to time lived in states of less than full health. These are responsible for 54% of all African DALYs and approximately half of all deaths. (0.41 MB TIF) [file pmed.1000293.s002.tif]

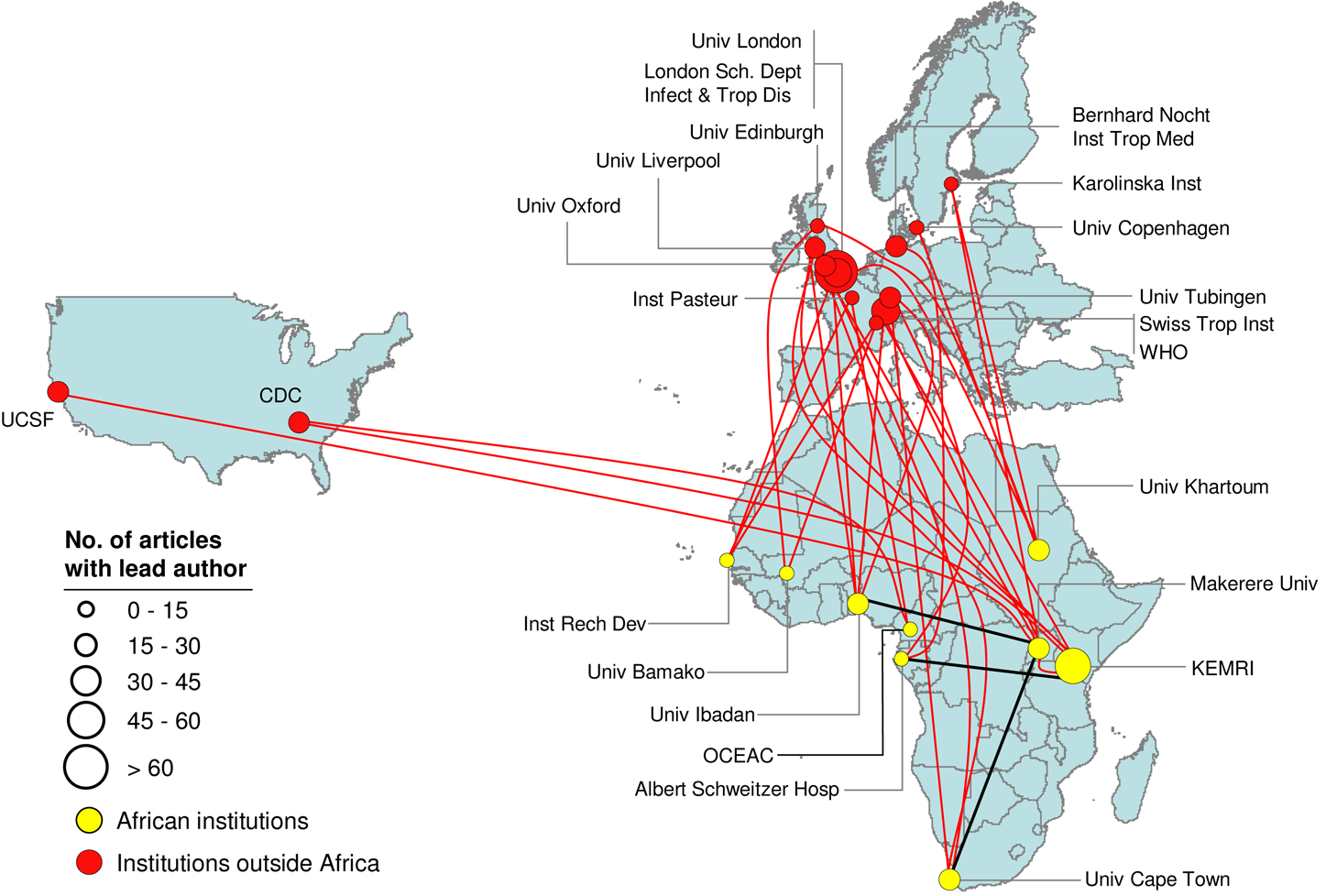

Supplement: Figure S3 — Collaboration bias towards the US and Europe for the malaria R&D network. Network mapping of most collaborative institutions leading articles identified as directly related to malaria and published from 2004 to 2008. There are nine top centers in Africa spread across the continent, except in the northern region. Few articles are published in collaboration between African centers, but these are active in collaborating with institutions in the Northern Hemisphere, mainly in Europe. Yellow circles indicate institutions in Africa; red circles mark institutions outside of Africa. Circle diameter indicates the count of malaria articles identified in the analyzed period. Only the top most-collaborative institutions in the network and the links to and from Africa among them are shown. (0.55 MB TIF) [file pmed.1000293.s003.tif]

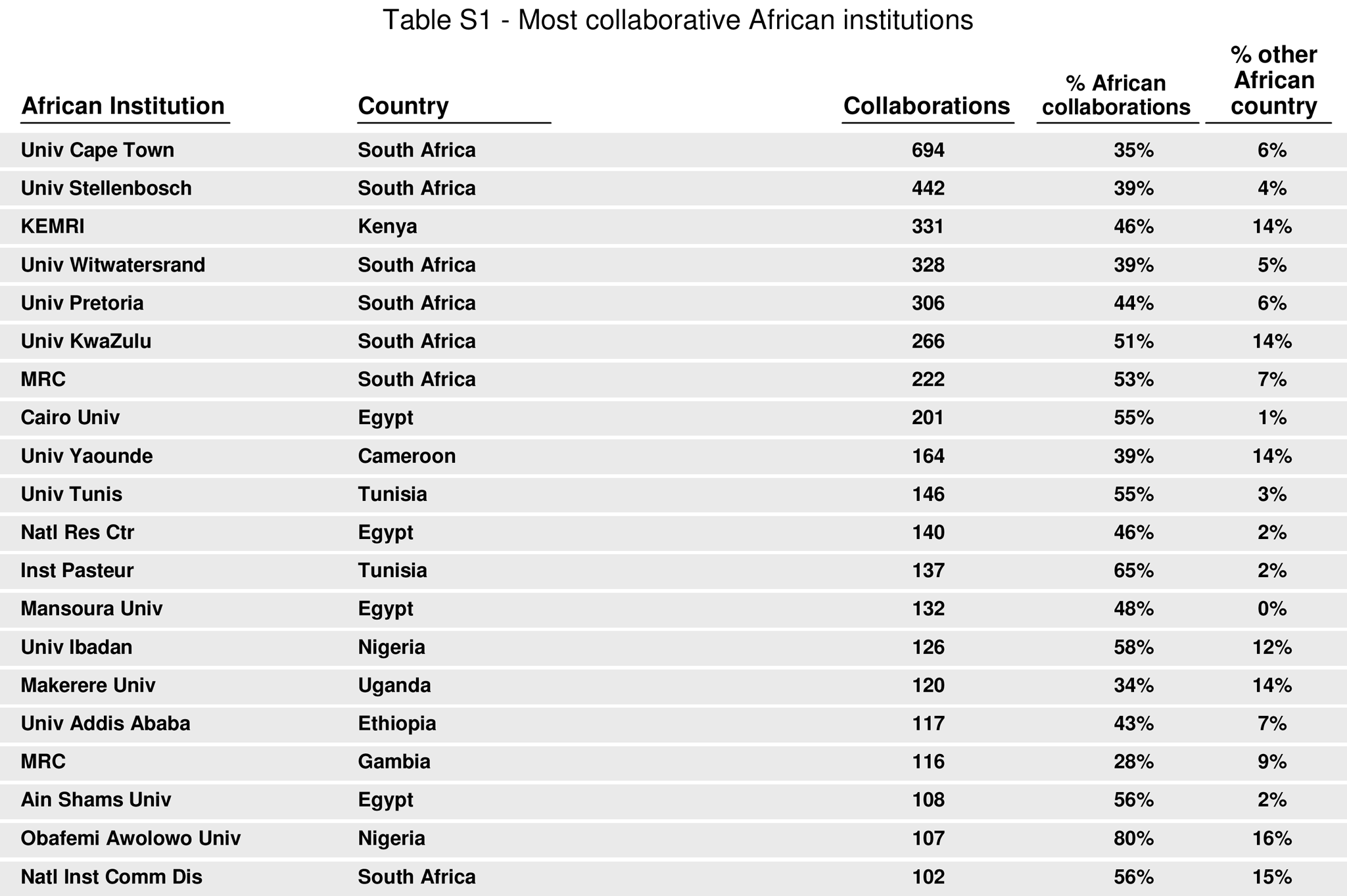

Supplement: Table S1 — Most-collaborative African institutions. (0.41 MB TIF) [file pmed.1000293.s004.tif]

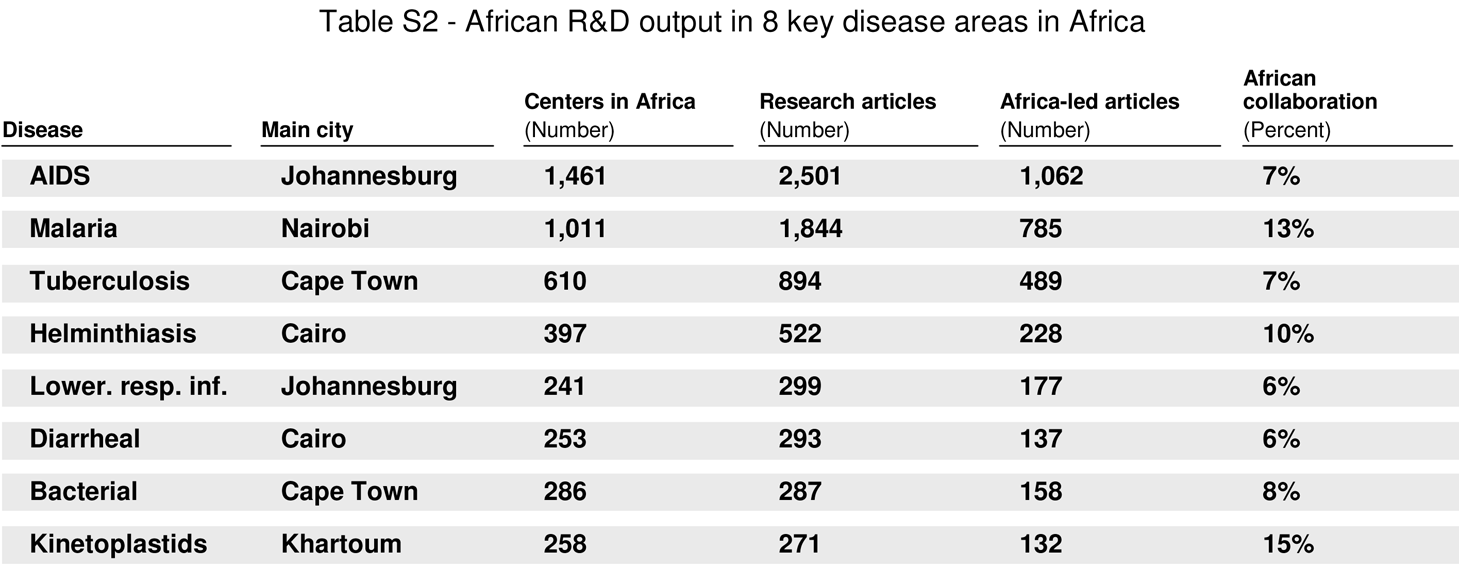

Supplement: Table S2 — African R&D output in eight key disease areas in Africa. (0.18 MB TIF) [file pmed.1000293.s005.tif]

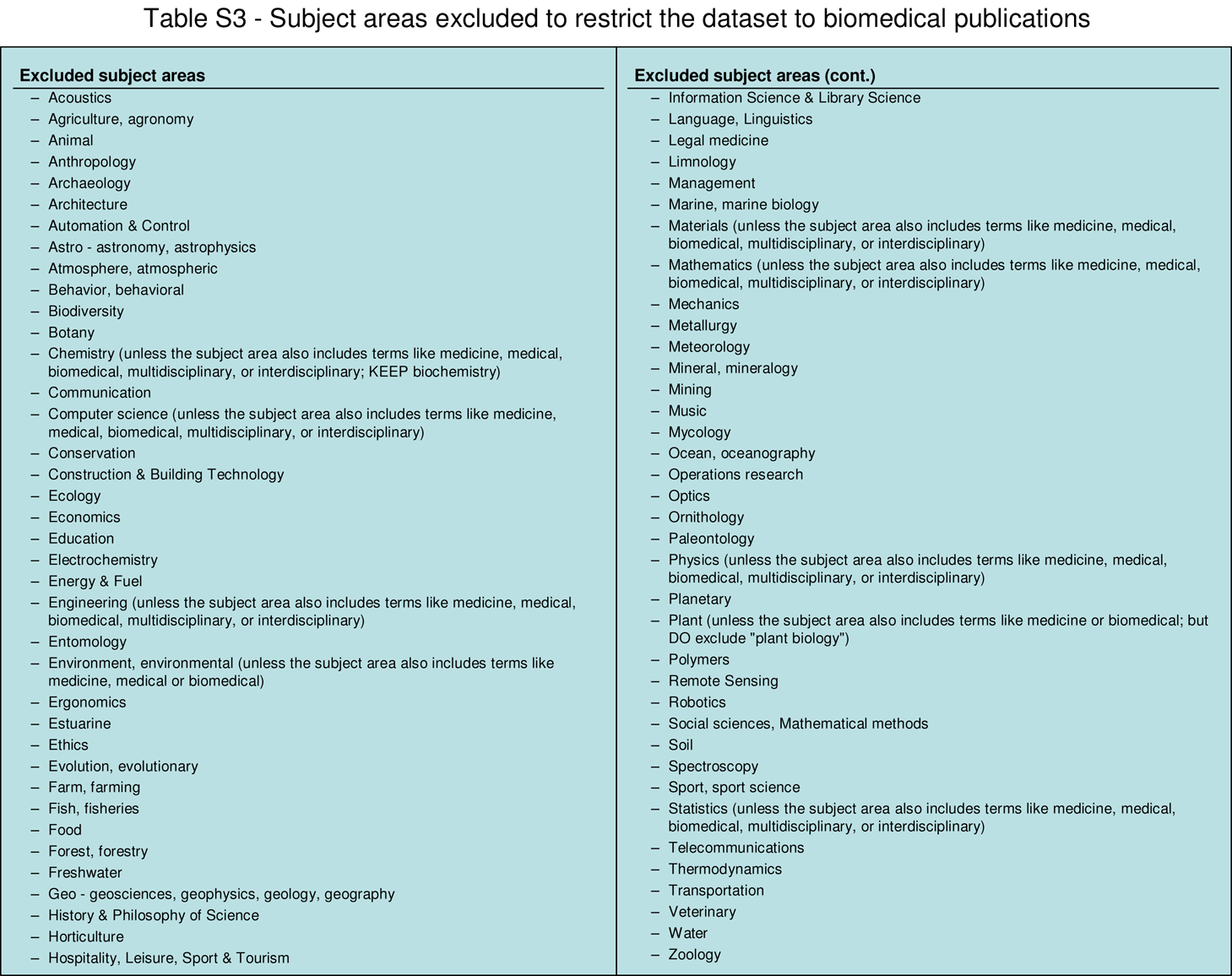

Supplement: Table S3 — Subject areas excluded to restrict the dataset to biomedical publications. (0.72 MB TIF) [file pmed.1000293.s006.tif]
