# Supplementary material for: Developing ANDI: A Novel Approach to Health Product R&D in Africa
Source: PLoS Med. 2010 Jun 29;7(6):e1000293. doi: 10.1371/journal.pmed.1000293 (PMC2893959; doi:10.1371/journal.pmed.1000293)
Supplement: Text S1 — Methodology. (0.03 MB DOC) [file pmed.1000293.s007.doc]

**Text S1 - Methodology**

**Data mining and database creation**

The database of Thomson Web of Science (TWS) was used to mine published article data due to its broad coverage of biomedical articles including relevant chemical and engineering literature as well as complete affiliation data for all authors in an article (<http://isiwebofknowledge.com/>). Other databases, including some publicly accessible ones such as PubMed were also consulted but the database of TWS provided a more comprehensive source of articles.

The affiliation field in the expanded SCI (Science Citation Index) database of TWS was queried for each of the 53 independent African countries. Only articles from 2004 to 2008 (5 years period) in all languages were retrieved. To focus our analysis on biomedical research we used the subject area field in Thomson to exclude certain non relevant subject areas, such as: basic chemistry, engineering, mathematics, and physics, animal behavior, botany, ecology, veterinary, zoology, environmental science and areas related to geology and mineralogy (Table S3 for full exclusion list). Importantly, interdisciplinary and biomedical chemistry, engineering, mathematics and physics were not excluded as they often contain relevant articles.

The data mining process resulted in 53 sets of text files, one for each African country represented (see Figure 2). These files were consolidated into a single master file of data and any duplicate records removed, resulting in 31,729 records representing published articles from 2004 to 2008. The master file was used to create 2 new databases designated “institutions database” and “articles database”.

The “institutions database” contains one record for every institution affiliated with each article (i.e., each article contributes a number of records equal to the number of institutions affiliated with the work). The fields in this database are: Article ID (Thomson 15-digit ISI number), Year of publication, Full address of the institution, Authors affiliated with the institution, Institution name, Institution city (city, suburb or village to which the postal code corresponds, or the name preceding the country name if there is no postal code), and Institution country.

The “articles database” contains one record per article with the following fields: Article ID, City of the corresponding address, Country of the corresponding address, Consolidated keywords field (author provided keywords + Thomson curated keywords), Journal, Country of publication, Number of unique institutions in affiliations, Number of authors, and Countries involved.

**Data analysis**

The institution name, city and country were used to create a unique identifier for each institution, which is automatically recognized in all records. Collaborative links were drawn between each institution in the corresponding address and all other institutions affiliated with the same article to create the network. As such, collaborations here reflect co-authorship of an article.

The same process was used to create the HIV/AIDS and the Malaria disease networks. A subset of research articles were identified for each disease by matching specific terms with the keywords present in the consolidated keywords field of the articles database (e.g., HIV, AIDS and immunodeficiency were the terms used to select for HIV/AIDS articles; while malaria, anopheles, and artemisinin were used for Malaria). The same process was used to identify the number of articles for each of the conditions listed in Figure 3. It should be noted that the consolidated keywords field contains author-provided keywords as well as keywords curated by Thomson Scientific. This set of keywords is likely to identify most articles pertaining to specific diseases like Malaria and HIV. This term-matching process was validated using an alternative procedure, i.e., using the full abstract of individual articles and no significant difference was found.

The “most collaborative” rankings to identify and map "hotspots" were based on the two most commonly used measures of network centrality: *degree* and *betweenness* [13, 14, 15, 16]. *Degree* is the simple count of links to other members of the network (collaborations). *Betweenness* is another measure of centrality in a network and refers to how many times a node lies in the shortest path connecting two other nodes that do not connect directly. Nodes with high *betweenness* and *degree* are central to the function of the network, and therefore considered hotspots. We used Ucinet (Analytic Technologies, USA) to calculate *degree* and *betweenness* [17].

*Degree* and *betweenness*, although related, are two distinct measures, and as such, the top institutions by each of the measures are highly overlapping but not the exact same. Only collaborative links going to and from Africa were mapped, i.e., links between institutions outside Africa were excluded. For clarity, only the top 20 institutions by *degree* and the top 20 by *betweenness* were plotted. As both top 20 sets are not exactly the same, the result is a number of institutions slightly over 20.

The volume of data generated and analysed automatically means that unforeseen institution or city spelling mistakes in the TWS database may result in the dual recognition of an institution or city (leading to underestimation of the number of collaborations and articles from that institution or city). Also the absolute number of institutional collaborations may be slightly overestimed. To mitigate the impact these potential errors have in our analysis, institutional and city spellings, including suburbs that contribute to a city’s output, were manually verified and the article and collaboration counts adjusted for the top 91 cities by output (Figure 3) and most collaborative institutions identified (Tables S1 and S2).
